# Complex traits and candidate genes: estimation of genetic variance components across multiple genetic architectures

**DOI:** 10.1093/g3journal/jkad148

**Published:** 2023-07-05

**Authors:** Mitchell J Feldmann, Giovanny Covarrubias-Pazaran, Hans-Peter Piepho

**Affiliations:** Department of Plant Sciences, University of California Davis, One Shields Ave, Davis, CA 95616, USA; International Maize and Wheat Improvement Center (CIMMYT), Carretera México-Veracruz, El Batán, 56130 Texcoco, Edo. de México, México; Biostatistics Unit, Institute of Crop Science, University of Hohenheim, Stuttgart 70599, Germany

**Keywords:** average semivariance, linear mixed model, variance component estimation, polygenic inheritance, oligogenic inheritance, Mendelian inheritance

## Abstract

Large-effect loci—those statistically significant loci discovered by genome-wide association studies or linkage mapping—associated with key traits segregate amidst a background of minor, often undetectable, genetic effects in wild and domesticated plants and animals. Accurately attributing mean differences and variance explained to the correct components in the linear mixed model analysis is vital for selecting superior progeny and parents in plant and animal breeding, gene therapy, and medical genetics in humans. Marker-assisted prediction and its successor, genomic prediction, have many advantages for selecting superior individuals and understanding disease risk. However, these two approaches are less often integrated to study complex traits with different genetic architectures. This simulation study demonstrates that the average semivariance can be applied to models incorporating Mendelian, oligogenic, and polygenic terms simultaneously and yields accurate estimates of the variance explained for all relevant variables. Our previous research focused on large-effect loci and polygenic variance separately. This work aims to synthesize and expand the average semivariance framework to various genetic architectures and the corresponding mixed models. This framework independently accounts for the effects of large-effect loci and the polygenic genetic background and is universally applicable to genetics studies in humans, plants, animals, and microbes.

## Introduction

Today, linear mixed models (LMMs) are routinely applied in plant breeding and quantitative genetics research. They are used for the prediction of genetic values in plants and animals (VanRaden 2008; Heffner *et al.* 2010; Meuwissen *et al.* 2016), or polygenic risk scores (PRSs) in humans (de Los Campos *et al.* 2013; Wray *et al.* 2019), to estimate the heritability of traits in target populations (Visscher *et al.* 2008; Legarra 2016), and to estimate ecological and evolutionary genetic parameters of behavioral traits (Ariyomo *et al.* 2013; Walsh and Lynch 2018). Genetic values are constructed from a combination of genetic effects; including Mendelian factors; which may have both additive and dominant sources of variance (Pincot *et al.* 2022), oligogenic factors consisting of few genetic factors and their epistatic interactions appropriate for marker-assisted prediction (MAP) (Tang *et al.* 2006), a polygenic term consisting of a dense genome-wide framework of markers assumed to have minor effects suitable for genomic prediction (GP); which may also account of additive and dominance sources of variance (Brandariz and Bernardo 2019), and a residual genetic term consisting of all genetic effects not accounted for by the previous genetic factors (Rutkoski *et al.* 2014; Rice and Lipka 2019; DeWitt *et al.* 2021). The ultimate objective in breeding applications is, typically, predicting the genotypic value, e.g. breeding value or genetic merit of a candidate individual (VanRaden 2008). For loci to provide actionable gains or diagnoses, they must explain a significant proportion of phenotypic and genetic variation in a population with alleles in segregation at target loci.

Candidate gene discovery through genome-wide association studies (GWAS) and quantitative trait locus (QTL) mapping is prolific in plant and animal populations (Lander and Schork 1994; Visscher *et al.* 2012, 2017). Despite decades of directional selection in many plant populations, loci impacting traits of interest still segregate, even in advanced breeding materials. These genome-wide analyses have implicated numerous genes and genomic regions in controlling a wide variety of simple and complex traits (Anderson *et al.* 2007; Septiningsih *et al.* 2009; Han *et al.* 2018; Demmings *et al.* 2019; Xin *et al.* 2020). However, the utility of such marker–trait associations may not be fully realized (Bernardo 2004, 2016). Large-effect and statistically significant loci typically only explain a fraction of the genetic and phenotypic variance in a population (Feldmann, Piepho, Bridges, *et al.* 2021), along with the polygenic fraction (Feldmann, Piepho, *et al.* 2022), except in extreme scenarios when Mendelian factors wholly control a trait.

Discovered loci rarely, if ever, explain 100% of the genetic variance, and understanding the multiple sources of variation and how they relate can help breeders and research prioritize targets and mitigate risk (Bernardo 2004, 2014). Genes with significant effects often dominate the “nonmissing heritability,” but they can mask or obscure the effects of other quantitatively acting genes and pleiotropically affect multiple quantitative phenotypes (Mackay 2001; Mackay *et al.* 2009; Eichler *et al.* 2010; De Villemereuil *et al.* 2018). For example, mutations in the *BRCA2* gene can have large effects but be incompletely penetrant, interact with other genes, and may be necessary but insufficient for predicting breast, ovarian, and other cancer risks in women (Gaudet *et al.* 2010). Accurately partitioning the Mendelian, oligogenic, and polygenic sources of variance allows researchers to assess the value conferred by specific loci.

Here, we use simulations to show that the average semivariance (ASV) provides accurate variance component estimates (VCEs) and variance component ratios for all relevant genetic terms regardless of study design or population type, e.g. outbred or inbred. We sought to provide a synthesis and extension of the previously published works on the ASV (Piepho 2019; Feldmann, Piepho, Bridges, *et al.* 2021; Feldmann, Piepho, *et al.* 2022) and to present a fully realized and efficient ASV approach for typical LMM analyses in human, plant, animal, and microbial genetics. We demonstrate how these models can be extended to handle more complex genetic structures, including adding multiple explanatory loci and marker–marker interactions, incorporating nonadditive dominance and epistasis variance, partitioning marker variance into additive and dominance components, and performing fully efficient stagewise analysis. To accommodate the models proposed in this research, we enabled the flexibility to provide the weights into the mixed model machinery in the form of a matrix (diagonal or nondiagonal) instead of a vector, which is now available in R/sommer>=v4.2.0. We provide examples of expressing the different models and extensions in the freely available R/sommer package (Covarrubias-Pazaran 2016). The ASV is a powerful tool for answering these questions regardless of the organism, population, or trait.

## Methods & materials

### Computer simulations model statements in R/sommer v4.2.0

We use computer simulations that follow the same style as in Feldmann, Piepho, Bridges, *et al.* (2021) and Feldmann, Piepho, *et al.* (2022) to demonstrate under fairly general conditions that ASV yields accurate estimates of variance components when (1) including main-effect loci alongside polygenic background, (2) partitioning additive and dominance sources of variance for single markers and polygene, and (3) performing fully efficient stagewise analyses.

#### Incorporating one target locus into GBLUP

LMM (1) is expressed as

mmer(fixed = Y ∼ 1,

  random = ∼ M +

        vsr(G, Gu = Kasv) +

        GR,

  rcov  = ∼ units,

  data  = data)

where data is an n×4 matrix containing the phenotypic observations *Y*, levels of the marker genotypes, entries, and levels of the residual genetic term, i.e. entries. The variable units is inferred by R/sommer::mmer() and can be considered as a column with as many levels as rows in the data (Covarrubias-Pazaran 2016).

The version of this model with $k_{M}$ embedded is expressed as

mmer(fixed = Y ∼ 1,

  random = ∼ vsr(M, Gu = KM) +

       vsr(G, Gu = Kasv) +

        GR,

  rcov  = ∼ units,

  data  = data)

where KM is the matrix $K_{M}=k_{M}^{-1}I_{n_{M}}$. All other variables are the same as previously defined.

We generated 18 experiment designs with different population sizes of n=500, 1,000, and 1,814, and number of clonal replicates per entry r=1, 2, and 4 for outbred H=0.38 and inbred H=0.0 populations. Clonal replicates are a particular case in plant genetics of hybrid (e.g. maize, rice, and sorghum) cropping systems and in clonally propagated species (e.g. strawberry, potato, and apple). In all examples, 100 populations are genotyped at m=5,000 loci. These 5,000 single nucleotide polymorphisms (SNPs) generated the purely additive polygenic background and one locus for the simple genetic effect. Marker genotypes, e.g. alleles, were drawn from a multivariate normal distribution to replicate the population structure of the 1,814 mice from Valdar *et al.* (2006) using R/MASS::mvrnorm() and transformed such that the population was heterozygosity H=0.38. We then estimated $K_{ASV}$ and excluded the targeted locus from the calculation of $K_{ASV}$. We also simulated residual genetic and residual effects each from a normal distribution with μ=0 and $\theta_{g_{R}}^{ASV}=\sqrt{50}$ and $\theta_{R}^{ASV}=\sqrt{40}$ using R/stats::rnorm(). A single explanatory locus was simulated with a segregation ratio of approximately 1:2:1 for AA:Aa:aa marker genotypes with μ=0 and $\theta_{m}^{ASV}=\sqrt{k_{M}\cdot 66}$ using R/stats::rnorm(). We simulated marker effects for all m=5,000 loci following a normal distribution μ=0 and $\theta_{g}^{ASV}=\sqrt{66}$, and each locus contributes equally. When multiplied by the centered marker genotypes and summed, the score is taken as each individual’s true additive genetic value *g*. For each simulated population we expressed LMM (1) using R/sommer::mmer() (Covarrubias-Pazaran 2016). In the second set of simulations, we used the same approach and the same mean and variance parameters. However, in this example, we simulated inbred lines in the background polygenic markers (H=0.0) and the foreground markers, e.g. 1:0:1 for AA:Aa:aa.

#### Incorporating multiple target loci into GBLUP

LMM (8) is expressed as

mmer(fixed = Y ∼ 1,

  random = ∼ M1 + M2 + M3 +

        M12 + M13 + M23 +

        M123 +

        vsr(G, Gu = Kasv) +

        GR,

  rcov  = ∼ units,

  data  = data)

where data is an n×10 matrix containing the phenotypic observations *Y*, seven columns corresponding to the marker effects and interactions, a factor-coding entries *G*, and a factor-coding levels of $g_{R}$.

Due to the similarities between our first set of experiments and this extension, we do not provide any additional simulations demonstrating the successes of this model extension. Feldmann, Piepho, Bridges, *et al.* (2021) demonstrated that multiple loci could be fit simultaneously with their interactions, and variance components can be estimated accurately. The same is true for models incorporating a polygenic genomic relationship matrix (GRM) as well. However, the user is encouraged to check that the higher order locus–locus interactions do not saturate the model and are not correlated with $K_{ASV}$.

#### Partitioning marker variance into additive and dominance components

LMM (9) is expressed as

mmer(fixed = Y ∼ 1,

  random = ∼ Ma + Md +

       vs(G, Gu = Kasv) +

        GR,

  rcov  = ∼ units,

  data  = data)

where data is an n×5 matrix containing the phenotypic observations *Y*, a factor-coding levels of $m_{A}$, a factor-coding levels of $m_{D}$, a factor-coding entries *G*, and a factor-coding levels of $g_{R}$. The factor coding of $m_{A}$ has three levels corresponding to AA:Aa:aa, and a factor coding of $m_{D}$ has two groups corresponding to the genetic state—either homozygous or heterozygous.

We performed one set of simulations for this model extension that follows the exact parameters as the first simulation set (m=5,000, n=500, H=0.38). In this simulation, we estimate which portion of the variance explained by a marker is from additive variance and which is from dominance variance. In this simulation, we estimate which portion of the additive genetic variance ($\theta_{g}^{ASV}=\sqrt{66}$), the marker explained variance by additive ($\theta_{m_{A}}^{ASV}=\sqrt{20}$) or dominance variance ($\theta_{m_{D}}^{ASV}=\sqrt{20}$), the residual genetic variance ($\theta_{g_{R}}^{ASV}=\sqrt{50}$), and the residual variance (on an entry-mean basis) ($\theta_{R}^{ASV}=\sqrt{40}$). In our simulations, 50% of the variance explained by the focal marker is from additive variation and 50% is dominance variation. The other parameters of the simulation are equal to the first set. We examined the accuracy of estimating each term as well as the accuracy of estimating the total variance explained by the focal marker.

#### Incorporating a genomic dominance relationship matrix into GBLUP

LMM (13) is expressed as

mmer(fixed = Y ∼ 1,

  random = ∼ M +

        vsr(Ga, Gu = Kasv) +

        vsr(Gd, Gu = Kasv˙D) +

       GR,

  rcov  = ∼ units,

  data  = data)

where data is an n×5 matrix containing the phenotypic observations *Y*, a factor-coding levels of the marker genotypes, and three equivalent factor-coding entries, to be used for the additive, dominance, and residual genetic terms.

We performed one set of simulations for this model extension that follows the exact parameters as the first simulation set (m=5,000, n=500, H=0.38). In this simulation, we estimate which portion of the polygenic variance is from additive ($\theta_{g_{A}}^{ASV}=\sqrt{33}$) or dominance ($\theta_{g_{D}}^{ASV}=\sqrt{33}$). In this simulation, the dominance polygenic variance is the same magnitude as the additive polygenic variance, and the other simulation parameters are equal to the first set. We also controlled the residual genetic variance ($\theta_{g_{R}}^{ASV}=\sqrt{50}$) and the residual variance (on an entry-mean basis) ($\theta_{R}^{ASV}=\sqrt{40}$), as in all simulations. We examined the accuracy of estimating each term.

#### Incorporating stagewise meta-analysis into GBLUP

LMM (15) for stage 1 is expressed as

mmer(fixed = Y ∼ G,

  random = ∼ Block,

  rcov  = ∼ units,

  data  = data)

where data is an n×3 matrix containing the phenotypic observations *Y*, one factor coding for the entry ID and one-factor coding for Blocks within the $n_{e}$ environment. Blocks and other within-location design elements can be incorporated as random effects using the random = syntax. In R/sommer, $\Sigma_{e}$ is obtained from each location as the ‘VarBeta‘ matrix in the R/sommer::mmer() output. “VarBeta” is the name of the model estimated variance–covariance matrix among entry means in R/sommer. The $\Sigma_{e}$s are then bound corner-to-corner, which is accomplished using R/sommer::adiag1() to obtain Ω. We then take the inverse of Ω using R/base::solve().

The LMM for stage 2 (17) is expressed as

mmer(fixed = Y2 ∼ Env - 1,

  random = ∼ vsr(M, Gu = KM) +

        vsr(G, Gu = Kasv) +

        G:Env + GR,

   rcov = ∼ vsr(units,

        Gti = matrix(invSigma2,1,1),

        Gtc = matrix(3,1,1)),

 nIters  = 25,

 emWeight = rep(1,25),

 W     = invOmega,

 data   = data)

where data is an n×5 matrix containing the adjusted entry means, or BLUEs, from stage 1 (Y2) a factor-coding levels of *M*, two equivalent factor-coding entries, e.g. *G* and $g_{R}$, and factor-coding environments *Env*. In this approach, we must fix the residual variance component equal to 1 so that all the scaling of the $invOmega=\Omega^{-1}$ is unaffected by the model estimation process. Within the vsr() argument, the Gti() and Gtc() arguments are used to set the initial value of the variance component equal to the inverse of the variance among adjusted entry means (invSigma2 = ${\hat{\sigma }}^{-2}$) and to constrain the variance component estimation to a fixed value by setting the first argument equal to 3 (Covarrubias-Pazaran 2023). In this example, we use 25 iterations of the 100% expectation-maximization (EM) algorithm; however, the EM and Newton-Raphson (NR) methods can be exchanged or averaged, i.e. average information, by changing the emWeight argument. This is not a general rule or recommendation. The large number of iterations we used caused this analysis to be computationally expensive and inefficient.

We performed one set of simulations for this model extension following the exact parameters of the first simulation set (m=5,000, n=500, H=0.38). In this simulation, we estimate which portion of the additive genetic variance ($\theta_{g}^{ASV}=\sqrt{66}$), the marker explained variance ($\theta_{m}^{ASV}=\sqrt{40}$), the residual genetic variance ($\theta_{g_{R}}^{ASV}=\sqrt{50}$), the genotype-by-environment interaction variance ($\theta_{GE}^{ASV}=\sqrt{90}$), and the residual variance (on an entry-mean basis) ($\theta_{\epsilon }^{ASV}=\sqrt{40}$). In this simulation, the dominance polygenic variance is the same magnitude as the additive polygenic variance, and the other simulation parameters are equal to the first set. We examined the accuracy of estimating each term.

## Results and discussion

### Candidate genes and complex traits

Bernardo (2014) was the first to propose an integration of MAP and GP. Since then, empirical studies have validated the methodology (Rutkoski *et al.* 2014; Spindel *et al.* 2016; Rice and Lipka 2019). In contrast, others have shown little-to-no improvement over GP (Li *et al.* 2015; Galli *et al.* 2020), suggesting that modeling significant markers can improve prediction accuracy only when markers explain a substantial portion of both genetic and phenotypic variance (Galli *et al.* 2020). With the high densities of genome-wide markers commonly assayed in gene finding studies, investigators often identify DNA markers tightly linked to a candidate or known causal genes as exemplified by diverse real-world examples (Hayes and Goddard 2001; Hayes *et al.* 2010; Jensen *et al.* 2012; Visscher *et al.* 2012, 2017; Li *et al.* 2021). The candidate marker loci are nearly always initially identified by genome-wide searches using sequential (marker-by-marker) approaches such as GWAS and QTL analysis. Following the discovery of statistically significant marker–trait associations from a marker-by-marker genome-wide scan, the natural progression would be to analyze single locus or multilocus genetic models where the effects of the discovered loci are simultaneously corrected for the effects of other discovered loci, e.g. polygenic variation (Stroup *et al.* 2018; Gbur *et al.* 2020).

A marker will not explain a large portion of variance if that marker does not have a large, detectable effect. Thus, markers that explain a large part of the genetic variance will be the most useful for MAP and other diagnostic techniques. For example, consider Fusarium race one wilt resistance in strawberry, which is conferred by a single dominant acting locus Fw1 (Pincot *et al.* 2022). This locus explains nearly 100% of the phenotypic and genetic variance, and the mean differences delineate resistant vs. susceptible genotypes. Thus there is almost no added benefit of a genome-wide sample of markers over the single-marker assay (*m*) for product delivery and germplasm improvement. While the variance explained is directly linked to the effect size, it is not a direct substitute. However, the random effect machinery allows researchers to obtain variance component estimates, and effect sizes (e.g. best linear unbiased predictors, BLUPs) simultaneously (Searle *et al.* 1992), eliminating the need for multiple statistical models to assess the variance explained and the effect size of a target locus. The BLUP procedure is directly applied in this model, so it is natural to use the same statistical machinery to estimate genome-estimated breeding values (GEBVs) by genomic best linear unbiased prediction (GBLUP) and the genetic effect of a locus.

As a point of contrast, yield in maize (*Zea mays*) is heritable, but no single locus explains any appreciable amount of phenotypic or genotypic variance (Heffner *et al.* 2009, 2010). To improve yield in maize, GP is potentially a more valuable approach because the researcher, or breeder, can predict the polygenic value (*g*) without relying on any particular locus but instead capturing variation of a genome-wide sample of markers. The more challenging scenario is the intermediate case in which a trait is controlled by both loci that are discernible from the polygenic background and a quantitative polygenic effect.

The ratio between the variance explained by the oligogenic and polygenic terms with the total genetic or phenotypic variance is likely a significant factor determining the cost–benefit of incorporating MAP, GP, or both into a breeding or diagnostic program. Modeling an individual locus can be advantageous when the proportion of the phenotypic and genetic variance explained by the locus is reasonably large and not partially captured by other markers in linkage disequilibrium (LD) with the target (Bernardo 2014; Rutkoski *et al.* 2014; Pincot *et al.* 2022). In this case, one could factor code a pseudomarker from multiple markers bracketing a QTL to capture the variance explained by that locus, assuming that SNPs used to define a QTL region are highly correlated and will not saturate a model’s effective degrees of freedom. Also, the targeted markers should not fit the marker effect size distribution assumptions used for the marker background, e.g. that all marker effects contribute equally to the genomic variance and are drawn from the same distribution (Habier *et al.* 2007; Endelman 2011; Morota and Gianola 2014) and should not be in high LD with a large number of other markers.

### The entry mean, not the observation, is the “phenotype”

We believe the “phenotype” is the entry mean for a given subdivision of environments, not the individual observations that constitute that entry mean. Our discussion here is primarily predicated on plants, but does not necessarily exclude other organisms, where replicate observations may be available per entry. In the words of Dr. Rex Bernardo, “…the main focus of quantitative genetics is on identifying candidates with the best genotypic value for a target population of environments” (Bernardo 2020). However, fine- or broad-scaled any subdivision is of a target population of environments or market segment, we argue that several environments must be sampled from each subdivision. Ultimately, an average across those environments will be used to communicate the value of an entry to a specific subdivision of target environments or to all target environments, if appropriate. These subdivisions may be defined by market segments, maturity zones, patterns of G×E, management strategies, geopolitics, and other elements of interest to a breeding or research program. The granularity of the entry mean is important since not all environments, micro or macro, or market segments can be considered equal, and severe genotype-by-environment interactions (G×E) may limit the information contained in the entry mean (Heslot *et al.* 2013; González-Barrios *et al.* 2019). Conceptualizing the phenotype as the entry mean should pose little practical consequence as stagewise analyses, common in GWAS and GP, explicitly express this idea (Dias *et al.* 2020; Pincot *et al.* 2020; Endelman 2022) and variance component ratios, such as the broad sense heritability ($H^{2}$), are often reported on an entry-mean basis (Bernardo 2020). This concept is also concordant with single-stage analyses incorporating all entries and subdivisions as main effects and the interaction, such as in product placement and other late-stage trials (Buntaran *et al.* 2020). Below, we show that ASV can be accurately applied in single-stage and stagewise analyses.

### LMM analysis and the ASV

The ASV estimator of total variance (Piepho 2019) and the variance of single markers and marker–marker interactions (Feldmann, Piepho, Bridges, *et al.* 2021) is half the average total pairwise variance of a difference between entries and can be decomposed into independent sources of variance, e.g. genetic and residual. In this article, we assume that researchers can replicate entries independently—as in clonally propagated or inbred crop species—or can collect repeated measures on entries (e.g. individuals, families, or strains)—as in humans and animals—and then estimate the least square means (LSMs), best linear unbiased estimators (BLUEs), or other adjusted entry means in the first stage of a stagewise analysis (Piepho *et al.* 2012; Damesa *et al.* 2017, 2019). For simplicity, we assume that the residual variance–covariance matrix, which can take many forms (Piepho 2019), is $R=I_{n}\sigma_{\epsilon }^{2}$, where *n* is the number of entries (e.g. individuals, accessions, genotypes, lines, or animals). In stagewise analysis, R is estimated in the first stage and therefore does not need to be re-estimated in the second stage. Instead, it is forwarded to the second stage by proper weighting.

The form of the LMM for this analysis assuming only one explanatory marker is


(1) $$\bar{y}=1\mu +Z_{m}m+Ig+Ig_{R}+\bar{\epsilon }$$

where $\bar{y}$ is the vector of LSMs with y∼N(1μ,V), μ is the population mean and the only fixed effect, $Z_{m}$ is the design matrix linking entry means to marker genotypes, m is the vector of random effects of the main-effects locus with $m∼N(0,I\sigma_{m}^{2})$, g is the vector of random additive genetic effects associated with the genome-wide framework of marker excluding m with $g∼N(0,K_{ASV}\sigma_{g}^{2})$, $g_{R}$ is the vector of random residual genetic term—the portion of the total genetic effect not accounted for by m or g—with $g_{R}∼N(0,I\sigma_{g_{R}}^{2})$, and $\bar{\epsilon }$ is the random residual term with $\bar{\epsilon }∼N(0,R)$. We use a pooled estimate of $\sigma_{\bar{\epsilon }}^{2}$ obtained from the first stage, so this term is known.

We then calculated $K_{ASV}$ as


(2) $$K_{ASV}=\frac{\bar{X}{\bar{X}}^{T}}{\left(n-1{)}^{-1}\;tr(\bar{X}{\bar{X}}^{T}\right)}$$

where $\bar{X}=PX$ is the mean-centered marker matrix, X is the marker matrix coded [−1,0,1] for [aa,Aa,AA] genotypes, $\bar{K}=\bar{X}{\bar{X}}^{T}$ is the realized genomic relationship or kinship matrix, $P=I-n^{-1}1_{n}1_{n}^{T}$ is the idempotent mean-centering matrix, and tr(⋅) is the trace.

The ASV definition of total variance from LMM (1) is


(3) $$\begin{array}{rl} \theta_{\bar{y}}^{ASV} & =(n-1{)}^{-1}\;tr(VP)\\ & =\theta_{m}^{ASV}+\theta_{g}^{ASV}+\theta_{g_{R}}^{ASV}+\theta_{\bar{\epsilon }}^{ASV} \end{array}$$

where $\theta_{\bar{y}}^{ASV}$ is the total phenotypic variance, V is the variance–covariance among LSMs, $\theta_{m}^{ASV}$ is the average semivariance of the simple genetic term, $\theta_{g}^{ASV}$ is the average semivariance of the polygenic term, $\theta_{g_{R}}^{ASV}$ is the average semivariance of the residual genetic term, and $\theta_{\bar{\epsilon }}^{ASV}$ is the average semivariance of the residuals.

The ASV definition of genomic variance is


(4) $$\begin{array}{rl} \theta_{g}^{ASV} & =(n-1{)}^{-1}\sigma_{g}^{2}\;tr(XX^{T}P)\\ & =\left[\frac{tr(\bar{K})}{n-1}\right]\sigma_{g}^{2} \end{array}$$

In general, we replace the unknown parameter values ($\sigma_{g}^{2}$) with their REML estimates (${\hat{\sigma }}_{g}^{2}$) to obtain the ASV estimates (${\hat{\theta }}_{g}^{ASV}$). Following this form, it is possible to extend LMM (1) to include dominance and epistatic sources of variance (see below).

The ASV definition of marker-associated genetic variance is


(5) $$\begin{array}{rl} \theta_{m}^{ASV} & =(n-1{)}^{-1}\sigma_{m}^{2}\;tr(Z_{m}Z_{m}^{T}P_{m})\\ & =\left[\frac{\left(n-n^{-1}\sum_{h=1}^{n_{m}}n_{g\;:\;m_{h}}^{2}\right)}{n-1}\right]\sigma_{m}^{2}\\ & =k_{m}{\hat{\sigma }}_{m}^{2} \end{array}$$

where $P_{m}=I-{n_{m}}^{-1}1_{n_{m}}1_{n_{m}}^{T}$ is the idempotent mean-centering marker genotype design matrix, $n_{m}$ is the number of marker genotypes, and $n_{g\;:\;m_{h}}$ is the number of entries nested in the *h*th marker genotype. We are factor-coding marker genotypes in these analyses and the marker genotypes are treated as discrete categorical values instead of continuous values (dosage). It is possible to extend this using the approach for multilocus models as in Equation (8), with and without marker–marker interactions, described in Feldmann, Piepho, Bridges, *et al.* (2021). Specifically, $\theta_{m}^{ASV}$ is the *total* variance explained by a marker and is analogous to the total genetic variance used to calculate broad sense heritability, not the additive genetic variance.

It is important to consider the relationship between the main effect of markers and marker–marker interactions and $K_{ASV}$. When markers are highly correlated—due to linkage disequilibrium (LD) or selection bias—the LMM framework will fail to accurately partition variance between two main effects, even if an estimator is “unbiased.” One possible strategy here is to create multilocus genotypes, e.g. AA.AA, AA.AB,…, BB.AB, BB.BB, from several SNPs defining a target QTL region. If LD is high in the region, there should be far fewer levels of the multilocus genotype than possible combinations. The same is true if the marker genotypes are highly correlated with the geometry of the $K_{ASV}$—the LMM framework will fail to accurately partition the variance between the oligogenic foreground and the polygenic background. One way to assess this is to examine the correlation between the first few eigenvectors of $K_{ASV}$ and the main-effect marker genotypes. If the correlation is large in magnitude, regardless of direction, the LMM will likely struggle to partition the variance components between the two terms accurately.

The ASV definition of the residual genetic variance is


(6) $$\begin{array}{rl} \theta_{g_{R}}^{ASV} & =(n-1{)}^{-1}\sigma_{g_{R}}^{2}\;tr(I_{n}I_{n}^{T}P)\\ & =\sigma_{g_{R}}^{2} \end{array}$$

Importantly, all terms are estimated on the same scale as the residual variance $\theta_{\epsilon }^{ASV}$ on an entry-mean basis. As with the marker variance, the residual genetic variance will not be accurately partitioned from the polygenic background as $K_{ASV}\to I$. While $K_{ASV}$ needs to have similar global features—$n^{-1}\;tr(K)=1$ and $n^{-2}\sum_{i}\sum_{j}K_{ij}=0$—it is important that $K_{ASV}\neq I$.

The ASV definition of the residual variance is


(7) $$\begin{array}{rl} \theta_{\bar{\epsilon }}^{ASV} & =(n-1{)}^{-1}\sigma_{\epsilon }^{2}\;tr(I_{n}I_{n}^{T}P)\\ & =\sigma_{\bar{\epsilon }}^{2} \end{array}$$

The residual variance $\sigma_{\bar{\epsilon }}^{2}$ is estimated in the first stage and the estimate is carried forward to the second stage.

Two crucial results from Piepho (2019) and Feldmann, Piepho, Bridges, *et al.* (2021) are that (1) the ASV variance component estimates for the total genetic variance from a simple model are equivalent to the REML variance components and (2) for REML estimates that are not ASV equivalent there are simple constants that can be applied post hoc to obtain ASV variance component estimates. Feldmann, Piepho, *et al.* (2022), and this article shows that some ASV variance components (e.g. additive genetic variance, a single marker variance) can conveniently be obtained by scaling the variance–covariance matrices for the specific random effects in the model directly.

### Simulations confirm that ASV yields accurate estimates of all genetic variance components and ratios

**Fig. 1.**
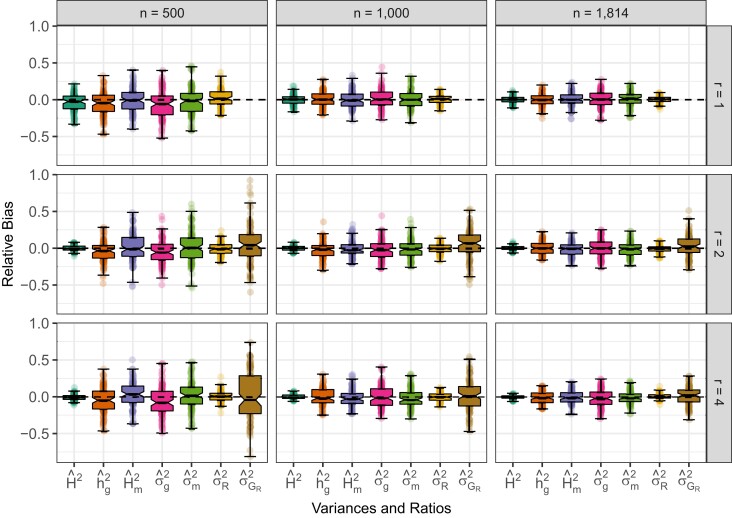
Effect of *n* and *r* on the relative bias of variance components and ratios in simulated outbred populations. Phenotypic observations were simulated for 100 samples with n=500, 1,000, and 1,814 (left to right) genotyped for m=5,000 SNPs and the average heterozygosity H=0.38. The relative bias of marker heritability, genomic heritability estimates (${\hat{h}}_{g}^{2}$), broad sense heritability, genomic variance, marker variance, residual genetic variance, and residual variance heritability when the number of replicates of each entry (*r*) =1 (upper panel), 2 (middle panel), and 4 (lower panel). Each box’s upper and lower halves correspond to the first and third quartiles (the 25th and 75th percentiles). The notch corresponds to the median (the 50th percentile). The upper whisker extends from the box to the highest value within 1.5×IQR of the third quartile, where *IQR* is the inter-quartile range or distance between the first and third quartiles. The lower whisker extends from the first quartile to the lowest value within 1.5×IQR of the quartile. The dashed line in each plot is the true value from simulations.

**Fig. 2.**
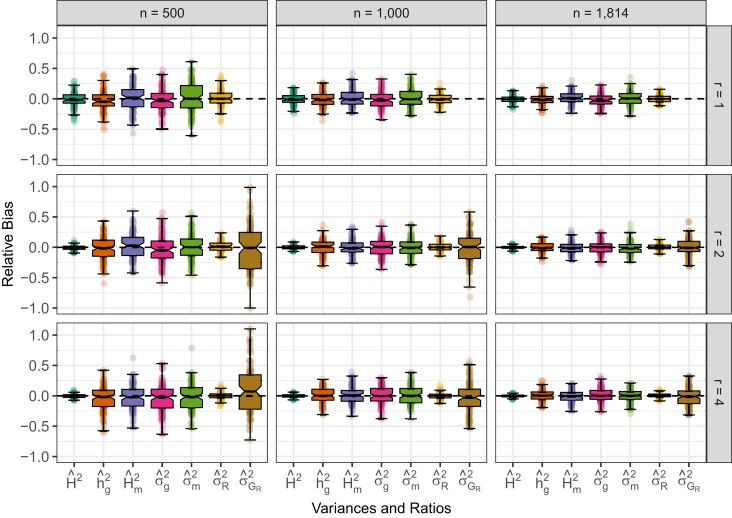
Effect of *n* and *r* on the relative bias of variance components and ratios in simulated inbred populations. Phenotypic observations were simulated for 100 samples with n=500, 1,000, and 1,814 (left to right) genotyped for m=5,000 SNPs and the average heterozygosity H=0. The relative bias of marker heritability, genomic heritability estimates (${\hat{h}}_{g}^{2}$), broad sense heritability, genomic variance, marker variance, residual genetic variance, and residual variance heritability when the number of replicates of each entry (*r*) =1 (upper panel), 2 (middle panel), and 4 (lower panel). Each box’s upper and lower halves correspond to the first and third quartiles (the 25th and 75th percentiles). The notch corresponds to the median (the 50th percentile). The upper whisker extends from the box to the highest value within 1.5×IQR of the third quartile, where *IQR* is the inter-quartile range or distance between the first and third quartiles. The lower whisker extends from the first quartile to the lowest value within 1.5×IQR of the quartile. The dashed line in each plot is the true value from simulations.

As shown in previous studies (Piepho 2019; Feldmann, Piepho, Bridges, *et al.* 2021; Feldmann, Piepho, *et al.* 2022), ASV is ideal for estimating the variance explained by both single loci and GRMs. In our simulations, we included variation in population size, e.g. n=500, 1,000, and 1,814, and replication of entries, e.g. r=1, 2, and 4 for both outbred (Fig. 1) and inbred populations (Fig. 2). We can see that the same pattern has emerged as in previous studies; the ASV approach yields accurate and consistent estimates of variance components and variance component ratios from LMM analyses regardless of the constitution of the population or the study design. Even when there is only one replicate per entry (r=1), all explanatory genetic terms are accurately partitioned from the total variance. As *n* increased from 500 to 1,814, the precision of estimates increased dramatically (the sampling variance decreased). Increasing *r* from 1 to 4 did not affect the precision or accuracy of genomic and marker-associated variances. However, increased numbers of replicates did improve the precision of residual variance components. This is because entries are replicated among plots (n⋅r), but markers and other genetic components are replicated among entries (*n*). Our simulations, in conjunction with our previous results (Piepho 2019; Feldmann, Piepho, Bridges, *et al.* 2021; Feldmann, Piepho, *et al.* 2022), demonstrate that in most populations—human, animal, plant, or microbe—the ASV will yield accurate and easily interpreted estimates of different variance components.

### LMM extensions incorporating the ASV

While an important model, LMM (1), only covers a narrow scope of the possible genetic models and experiments, we want to provide researchers with a clear strategy for expanding this approach to more complex systems. This section demonstrates how to partition the additive and dominance variance from a single marker, incorporate multiple explanatory loci, their interactions into the model, and nonadditive polygenic terms, and achieve fully efficient stagewise analysis. Depending on the population, trait, environment, etc., the unique components of the models demonstrated here can be combined to accurately and holistically decompose the multitude of potential sources of genetic variation. The code to execute these models using the R/sommer>=v4.2.0 (Covarrubias-Pazaran 2016) is provided in the *Methods & Materials* section.

#### Extension #1: Incorporating multiple target loci and locus–locus interactions

It is common for multiple QTL to be implicated from genetic studies (Rutkoski *et al.* 2014; Lopdell *et al.* 2019; Rice and Lipka 2019), the utility of which is not always certain (Bernardo 2001, 2004). While the simulations in this paper rely exclusively on LMM (1), this model can be easily expanded to include multiple explanatory loci and their interactions or statistical epistasis (Álvarez-Castro and Carlborg 2007), as demonstrated by Feldmann, Piepho, Bridges, *et al.* (2021). For example, the LMM with three main-effect loci is


(8) $$\begin{array}{rl} \bar{y} & =1_{n}\mu +\sum_{i\;=\;1}^{3}Z_{m_{i}}m_{i}+\sum_{i=1}^{2}\sum_{\;j=i+1}^{3}Z_{m_{ij}}m_{ij}\\ & +Z_{m_{123}}m_{123}+Ig+Ig_{R}+\bar{\epsilon } \end{array}$$

where $m_{i}$ is the random effect of the *i*th main-effect marker, $m_{ij}$ is the random effect of the two-way interaction between the *i*th and *j*th markers, and $m_{123}$ is the random effect of the three-way interaction between the three main-effect loci. $Z_{m_{i}}$, $Z_{m_{ij}}$, and $Z_{m_{123}}$ are design matrices that link levels of the explanatory marker and interactions to LSMs in y. The rest of the terms have the same definitions. LMM (8) follows directly from Equation (1) and the results from Feldmann, Piepho, Bridges, *et al.* (2021), specifically the two and three loci examples.

Since we are factor-coding marker genotypes in these models, that is we are thinking of the marker genotypes as discrete categorical values instead of continuous values (dosage), it is possible to fully saturate the multilocus interaction with more levels than are observed in a given data set. Hence, it is important to consider the number of interaction terms evaluated. In this situation, packages such as lme4::lmer() will report an error that the “number of levels of each grouping factor must be < number of [LSMs]” (Bates *et al.* 2015). Further, these models assume that random effects are independent, so we do not advise incorporating main effects from the SNPs used to define a target QTL region. Instead, it is possible to factor code a pseudohaplotype from the best markers bracketing a QTL to capture the variance explained by that locus, which can be more informative than a single SNP. This approach assumes that SNPs used to define a QTL region are not independent and do not fully saturate the model.

#### Extension #2: Partitioning $\theta_{m}^{ASV}$ into additive ($\theta_{m_{A}}^{ASV}$) and dominance ($\theta_{m_{D}}^{ASV}$) components

The factor-coding of the Mendelian and oligogenic markers is a different approach than is standard in GWAS. In GWAS, markers are typically treated as fixed and coded as continuous values, e.g. the dosage model. Assuming that a researcher is working with an outbred species and the heterozygosity (*H*) ≠0, the dominance variance can be significant, and partitioning the additive and dominance sources of variance from significant markers can be useful in hybrid crop breeding and disease risk prognoses. Our goal is to partition $\theta_{m}^{ASV}$, the variance explained by a focal locus, into its additive ($\theta_{m_{A}}^{ASV}$) and dominance ($\theta_{m_{D}}^{ASV}$) components.

Here, we demonstrate an LMM that can partition the main-effect marker’s additive and dominance sources of variance by transforming the marker genotypes into two factors. The form of the linear mixed model (LMM) for this analysis assuming only one explanatory marker is


(9) $$\bar{y}=1\mu +Z_{m_{A}}m_{A}+Z_{m_{D}}m_{D}+Ig+Ig_{R}+\bar{\epsilon }$$

where $m_{A}$ is the random additive effect of the main-effect locus with $m_{A}∼N(0,I\sigma_{m_{A}}^{2})$ and $m_{D}$ is the random dominance effect of the main-effect locus with $m_{D}∼N(0,I\sigma_{m_{D}}^{2})$. $Z_{m_{A}}$ is an n×3 design matrix linking marker genotypes to LSMs and $Z_{m_{D}}$ is an n×2 design matrix linking genotypic state, either homozygous (*AA* and *aa*) or heterozygous (*Aa*), to LSMs. For example, the $Z_{m_{A}}$ and $Z_{m_{D}}$ design matrices for five individuals (rows) with marker genotypes at a focal locus of [AA,Aa,Aa,aa,aa]=[1,0,0,−1,−1] are


(10) $$Z_{m_{A}}=\left[\begin{array}{ccc} 1 & 0 & 0\\ 0 & 1 & 0\\ 0 & 1 & 0\\ 0 & 0 & 1\\ 0 & 0 & 1 \end{array}\right],\;Z_{m_{D}}=\left[\begin{array}{cc} 1 & 0\\ 0 & 1\\ 0 & 1\\ 1 & 0\\ 1 & 0 \end{array}\right]$$

Other terms are defined in LMM (1). This extension is a partition of Equation (1). So we expect that Equations (1) and (9) are equivalent, except that Equation (9) will yield a variance component for each of the additive and dominance terms, while Equation (1) only yield the total genetic variance.

The ASV estimate of the additive variance explained by a locus is obtained as in Equation (5) by


(11) $$\begin{array}{rl} {\hat{\theta }}_{m_{A}}^{ASV} & =(n-1{)}^{-1}{\hat{\sigma }}_{m_{A}}^{2}\;tr(Z_{m_{A}}Z_{m_{A}}^{T}P_{m_{A}})\\ & =\left[\frac{n-n^{-1}\sum_{h=1}^{n_{m_{D}}}n_{g\;:\;m_{A_{h}}}^{2}}{n-1}\right]{\hat{\sigma }}_{m_{A}}^{2} \end{array}$$

where $P_{m_{A}}=I-n_{m_{A}}^{-1}1_{n_{m_{A}}}1_{n_{m_{A}}}^{T}$, $n_{m_{A}}$ are the number of levels coding the marker additive effects, $n_{g\;:\;m_{A_{h}}}$ is the number of entries nested in the *h*th marker genotype (Feldmann, Piepho, Bridges, *et al.* 2021). The average semivariance estimate of the dominance variance explained by a locus is obtained by


(12) $$\begin{array}{rl} {\hat{\theta }}_{m_{D}}^{ASV} & =(n-1{)}^{-1}{\hat{\sigma }}_{m_{D}}^{2}\;tr(Z_{m_{D}}Z_{m_{D}}^{T}P_{m_{D}})\\ & =\left[\frac{n-n^{-1}\sum_{\;j=1}^{n_{m_{D}}}n_{g\;:\;m_{D_{\;j}}}^{2}}{n-1}\right]{\hat{\sigma }}_{m_{D}}^{2} \end{array}$$

where $P_{m_{D}}=I-n_{m_{D}}^{-1}1_{n_{m_{D}}}1_{n_{m_{D}}}^{T}$, $n_{m_{D}}$ are the number of levels coding the genetic status, e.g. homozygous or heterozygous, $n_{g\;:\;m_{D_{i}}}$ is the number of entries nested in the *j*th genetic state. The sum of $[k_{m_{A}}{\hat{\sigma }}_{m_{A}}^{2}+k_{m_{D}}{\hat{\sigma }}_{m_{D}}^{2}]=[{\hat{\theta }}_{m_{A}}^{ASV}+{\hat{\theta }}_{m_{D}}^{ASV}]={\hat{\theta }}_{m}^{ASV}$ and $[{\hat{\theta }}_{m_{A}}^{ASV}+{\hat{\theta }}_{m_{D}}^{ASV}]-{\hat{\theta }}_{m}^{ASV}=2.21\times 10^{-5}$. ${\hat{\theta }}_{m}^{ASV}$ is an accurate and consistent estimate of the variance explained by a marker (Feldmann, Piepho, Bridges, *et al.* 2021). The likelihood ratio (LR) between LMM (1) and (9) was LR≈0. It was not significant in any simulated populations ($P_{LR}>0.2$), suggesting that there is no appreciable difference between the model likelihood of Equations (1) and (9). For each term, ${\hat{\theta }}_{m_{A}}^{ASV}$ and ${\hat{\theta }}_{m_{D}}^{ASV}$, the average bias’ across the 100 simulated populations was 1.06% and −1.24%, respectively.

#### Extension #3: Incorporating additional polygenic terms for genome-wide dominance ($g_{D}$)

LMM (1) can also be extended to include both additive ($g_{A}$) and dominance ($g_{D}$) sources of genomic variance (Vitezica *et al.* 2013, 2017; Zhang *et al.* 2021). The form of the LMM for analysis with both $g_{A}$ and $g_{D}$ assuming only one explanatory marker *M* is


(13) $$\bar{y}=1\mu +Z_{m}m+Ig_{A}+Ig_{D}+Ig_{R}+\bar{\epsilon }$$

where $g_{A}$ and $g_{D}$ are random effect vectors for the additive and dominance polygenic effects, respectively, with $g_{A}∼N(0,K_{ASV}\sigma_{g_{A}}^{2})$ and $g_{D}∼N(0,K_{ASV}^{D}\sigma_{g_{D}}^{2})$. The ASV dominance kernel is


(14) $$K_{ASV}^{D}=\frac{\bar{W}{\bar{W}}^{T}}{\left(n-1{)}^{-1}\;tr(\bar{W}{\bar{W}}^{T}\right)}$$

where W=1−|X|, assuming X is coded [−1,0,1], and $\bar{W}=PW$. This is a feasible approach to improve genetic performance in crossbred populations with large dominance genetic variation (Nishio and Satoh 2014; Vitezica *et al.* 2017; Xiang *et al.* 2018). Both $K_{ASV}$ and $K_{ASV}^{D}$ have the matrix properties proposed by Speed and Balding (2015); i.e. $n^{-1}\;tr(K)=1$ and $n^{-2}\sum_{i}\sum_{j}K_{ij}=0$. The dominance variance estimated with $K_{ASV}^{D}$ was accurate, and the relative bias from 100 simulated populations was −3.32%. Interestingly, $K_{ASV}^{D}$ is substantively different than both of the matrices proposed by Nishio and Satoh (2014) and Su *et al.* (2012).

Feldmann, Piepho, *et al.* (2022) showed that, regardless of population quality, a GRM with an average diagonal value of 1 and an average element value of 0 will produce consistent variance component estimates of the genomic variance. A matrix with the same properties calculated from a dominance coding will produce similarly unbiased parameter estimates. The dominance GRM proposed by Su *et al.* (2012) has an average diagonal value of 1, but the average element value is >0, leading to a systematic underestimating since the covariances are overestimated. The dominance GRM proposed by Nishio and Satoh (2014) has an average element value of 0, but the average element value is <1, leading to a systematic overestimating since the variances (diagonals) are underestimated. This is true for a wide range of population heterozygosities.

#### Extension #4: Stagewise LMM analysis for multienvironment trials (METs) and meta-analysis

Stagewise analyses are common in plant breeding trials in academic studies and the seed industry (Damesa *et al.* 2017, 2019). One reason for this is that plant breeders are often not interested in the performance per se of a line or hybrid *within* a specific location unless the presence of cross-over (e.g. rank change) G×E is large enough to make data from one target environment uninformative in another set of target environments. Instead, plant breeders are often more interested in the performance of entries *across* environments (Bernardo 2020). It is common then to fit a first model that accounts for the variation of random design elements, e.g. locations, years, blocks, and fixed genotype effects, to obtain the phenotype—estimated marginal means (EMMs) or best linear unbiased estimators (BLUEs)—for use in subsequent analyses. In subsequent stages, these entry-means *within* environments in a subdivision are used as the response variable.

**Fig. 3.**
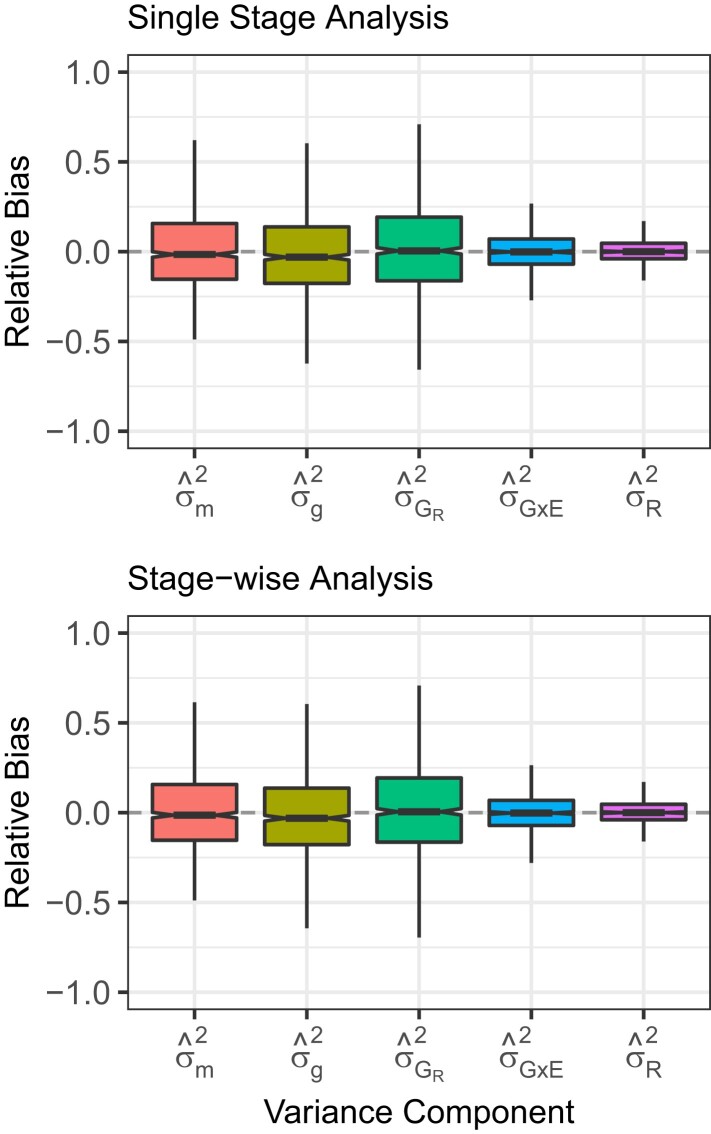
Comparison of VCEs estimated from single-stage and stagewise analyses of 500 entries replicated in four environments with block effects. The relative bias of genomic variance (${\hat{\sigma }}_{g}^{2}$), marker variance (${\hat{\sigma }}_{m}^{2}$), residual genetic variance (${\hat{\sigma }}_{g_{R}}^{2}$), genotype-by-environment interaction variance (${\hat{\sigma }}_{G\times E}^{2}$), and residual variance (${\hat{\sigma }}_{R}^{2}$) analyzed in a single stage (upper panel) or stagewise stages (lower panel). Each box’s upper and lower halves correspond to the first and third quartiles (the 25th and 75th percentiles). The notch corresponds to the median (the 50th percentile). The upper whisker extends from the box to the highest value within 1.5×IQR of the third quartile, where *IQR* is the inter-quartile range or distance between the first and third quartiles. The lower whisker extends from the first quartile to the lowest value within 1.5×IQR of the quartile. The dashed line in each plot is the true value from simulations.

In general, the single-stage analysis, when performed correctly, should be considered the “gold standard.” However, there are experimental conditions where the stagewise analysis may be simpler to execute and functionally equivalent to the single-stage analysis when performed correctly (Fig. 3), and, given the frequency of naive stagewise analyses—those that fail to incorporate the variance–covariance matrix of entry means, or even appropriate weights, from stage 1 into the second stage—we felt it prudent to highlight the simplicity of these approaches to a general audience. The purpose here is not to convince the reader that multistage analyses are superior (they are not), nor to provide a one-size-fits-all solution for every experiment (that is impossible), but to provide a path for users to accomplish fully efficient, multistage analyses using free, open-source software. Generally, the stagewise analysis should be considered a possible backup to the single-stage analysis, not the standard (Schulz-Streeck *et al.* 2013; Gogel *et al.* 2018; Damesa *et al.* 2019; Buntaran *et al.* 2020).

The LMM for stage one is


(15) $$y_{e}=X_{e}g_{e}^{*}+Z_{e}u_{e}+\epsilon_{e}$$

where $X_{e}$ is the fixed effect design matrix linking observations to entries, and $g_{e}^{*}$ are the fixed effects (e.g. BLUEs) for the entries in the *e*th environment, $Z_{e}$ is the random effect design matrix for design (e.g. blocks) elements within each environment (e.g. years and locations), and $\epsilon_{e}$ are the residuals and $\epsilon_{e}∼N(0,R_{e})$, where $R_{e}$ is the residual variance–covariance matrix estimated in the *e*th environment. This model is fit within each environment independently.

From these models, we obtain the adjusted entry means $\bar{y}$ and the variance–covariance matrices of the entry means $\Sigma_{e}$ from each of $n_{e}$ environments, where $n_{e}$ are the number of environments. We can then construct the $n_{ge}\times n_{ge}$ block-diagonal stage one Ω matrix as in Equation (16).


(16) $$\Omega_{n_{ge}}=\left[\begin{array}{ccc} \Sigma_{1} & \cdots & 0\\ \vdots & \ddots & \vdots \\ 0 & \cdots & \Sigma_{n_{e}} \end{array}\right]$$

where $n_{ge}$ is the number of entries nested in environments; for example, if there are 500 entries in four environments, $n_{ge}=2,000$. This method allows us to carry the full $\Omega_{n_{ge}}$ over from stage one to stage two of the analysis.

The second stage can take several forms with varying complexities, more complete approximations of $\Omega_{n_{ge}}$ or $\Omega_{n_{ge}}^{-1}$, and software accessibility. Briefly:

1. *In a naive stagewise analysis*, the residual matrix is given as the identity matrix multiplied by a scalar ($\tilde{\Omega }=I_{n_{ge}}\omega^{-1}=I_{n_{ge}}\sigma^{2}$), where ω is a scalar ($\sigma^{-2}$) estimated by the second stage LMM, assuming that the variances for entry means are identical with 0 covariances (independent); IID. This approach is very common in plant sciences because it is simple but problematic outside a specific set of unrealistic conditions, i.e. IID entry means. It is simple because it does not require any information on precision from stage 1. It is problematic because the residual and genotype-by-environment variances are confounded. The naive approach does not require additional arguments for LMM software and can be executed in any LMM software.
2. *In a weighted stagewise analysis*, the $\tilde{\Omega }=D(\omega {)}_{n_{ge}}^{-1}$ matrix is diagonal, but each diagonal element may differ based on data-driven weight ($D(\omega {)}_{n_{ge}}$), where $D(\omega {)}_{n_{ge}}$ is an $n_{ge}$-dimension diagonal matrix, estimated in the first stage of the analysis. Importantly, these weights are derived as one of many possible diagonal approximations of $\Omega_{n_{ge}}$ (Móhring and Piepho 2009) or its inverse, e.g. $\Omega_{n_{ge}}^{-1}$, from the first stage of the analysis (Smith *et al.* 2001). The weighted approach may take multiple forms that may or may not neglect the covariances among entry mean, leading to discrepancies between the single-stage and stagewise analyses (Smith *et al.* 2001, 2005; Móhring and Piepho 2009). This approach requires an additional argument in LMM software, typically “weights,” which is input as a vector corresponding to entry means and internally transformed into a diagonal matrix, and can be executed in several free or paid software (Inc. 2013; Covarrubias-Pazaran 2016; Butler 2021).
3. *In a fully efficient stagewise analysis*, entry means are allowed to have nondiagonal covariance structures with $\tilde{\Omega }=\Omega_{n_{ge}}$, where $\Omega_{n_{ge}}$ is the full variance–covariance matrix of entry means from the different environments [defined in Equation (16)]. This approach is the most general solution for implementing stagewise meta-analyses, maintaining all variances and covariances without approximation, but it is the most limited in terms of software implementations. The full variance–covariance matrix of the entry means will be nondiagonal in most cases, and a diagonal matrix (weighted or unweighted) is almost invariably an approximation as the random main effects of the environment, or block, will induce a positive covariance among all entry means. Incorporating the full variance–covariance matrix requires a lot of additional data and significantly reduces the computational efficiency of the LMM, which may outweigh potential practice benefits. This approach requires the inverse of the full variance–covariance matrix, $\Omega_{n_{ge}}^{-1}$, as an input argument and can now be executed in R/sommer>=v4.2.0 (Covarrubias-Pazaran 2016).

The LMM for stage two is then:


(17) $$\bar{y}=1\mu +Xe+Z_{m}m+Z_{g}g+Z_{g_{R}}g_{R}+Z_{GE}g_{GE}+\bar{\epsilon }$$

where $\bar{y}$ are the adjusted entry means from stage one, μ is the population mean, X is the fixed effect design matrix linking environments to adjusted entry means, e are the fixed environmental main effects, g is the random additive genetic effect associated with the genome-wide framework of marker excluding m with $g∼N(0,K_{ASV}\sigma_{g}^{2})$, $g_{R}$ is the random residual genetic term—the portion of the total genetic effect not accounted for by m or g—with $g_{R}∼N(0,I_{n}\sigma_{g_{R}}^{2})$, $g_{GE}$ is the genotype-by-environment interaction term with $g_{GE}∼N(0,I_{n_{ge}}\sigma_{g_{GE}}^{2})$, and $\bar{\epsilon }$ is the structured residual term from stage one with $\bar{\epsilon }∼N(0,\Omega )$ With this model, we can estimate the breeding values across environments with marker information (K) as in GBLUP and can perform GWAS by adding an iterative term for single marker regression, such as $\sum_{i=1}^{\;j}\beta_{i}x_{i}$ where *j* is the number of markers, $\beta_{i}$ is the linear regression coefficient of the *i*th marker, and $x_{i}$ is the numeric coding of the *i*th markers genotypes, e.g. [−1,0,1].

We created 1,000 simulated populations with 1,000 entries and 5,000 markers using a similar approach to the other simulations in this experiment. However, we included Environmental and Block within Environment effects in this experiment. We estimate the variance explained by the polygenic background, a large-effect locus, the residual genetic variance, the genotype-by-environment interaction variance, and the nongenetic residual. The single stage analysis yielded relative biases of −1.55%, −3.04%, −0.45%, −0.12%, and 0.03% for the marker variance (${\hat{\sigma }}_{m}^{2}$), genomic variance (${\hat{\sigma }}_{g}^{2}$), residual genetic variance (${\hat{\sigma }}_{g_{R}}^{2}$), genotype-by-environment interaction variance (${\hat{\sigma }}_{g_{GE}}^{2}$), and residual variance (${\hat{\sigma }}_{\bar{\epsilon }}^{2}$), respectively (Fig. 3). The two stage analysis yielded relative biases of −1.39%, −3.09%, 0.48%, −0.21%, and 0.03% for the marker variance (${\hat{\sigma }}_{m}^{2}$), genomic variance (${\hat{\sigma }}_{g}^{2}$), residual genetic variance (${\hat{\sigma }}_{g_{R}}^{2}$), genotype-by-environment interaction variance (${\hat{\sigma }}_{g_{GE}}^{2}$), and residual variance (${\hat{\sigma }}_{\bar{\epsilon }}^{2}$), respectively (Fig. 3).

#### Extension #5: Incorporating $k_{M}$ directly into LMM analyses

Feldmann, Piepho, Bridges, *et al.* (2021) introduced $k_{M}$ (5) as a post hoc adjustment of the REML estimated variance explained by a marker to obtain ASV equivalent VCEs. This led to Feldmann, Piepho, *et al.* (2022), who showed that ASV estimates of the genomic variance could be obtained by scaling the genomic relationship before or after the LMM analysis and introduced $K_{ASV}$, eliminating the need for any post hoc adjustment. Scaling variance components a priori is not novel and is routine in genomic evaluation across species (VanRaden 2008; Astle and Balding 2009; Yang *et al.* 2010; Endelman and Jannink 2012; Legarra 2016; Vitezica *et al.* 2017). LMMs can directly scale the variance–covariance matrix for large-effect loci *M* by $k_{M}$.

Instead, if we define:


(18) $$K_{M}=k_{M}^{-1}I_{n_{M}}$$

where $K_{M}$ is $n_{M}\times n_{M}$ and $n_{M}$ is the number of marker genotypes at a given locus, we can essentially think of $K_{M}$ as a genomic relationship matrix, e.g. $K_{ASV}$, except that we apply $K_{M}$ to the levels of the marker genotype instead of entries.

The form of the LMM for this analysis assuming only one explanatory marker is the same as Equation (1), but where m is the random effect of the main-effect locus with $m∼N(0,K_{M}\sigma_{m}^{2})$. With this approach, we maintain the levels of the factor come from the same variance and zero covariance, but our scaling factor is embedded directly in the model eliminating the need for adjustment. Embedding $k_{M}$ in the LMM analysis using $K_{M}$ is equivalent to the post hoc adjustment that was proposed in Feldmann, Piepho, Bridges, *et al.* (2021), and so it is up to the user to determine which approach they prefer.

## Conclusions

ASV is a strategy that can be used for estimating and partitioning the total variance into components (Piepho 2019), such as the variance explained by loci and locus–locus (Feldmann, Piepho, Bridges, *et al.* 2021) and the genomic variance (Feldmann, Piepho, *et al.* 2022). The approach we are suggesting shares some common threads with the current thinking in quantitative genetics, particularly as it relates to genomic relatedness, genomic heritability, and GP (VanRaden 2008; Kang *et al.* 2010; Yang *et al.* 2010; Habier *et al.* 2013) but it also deviates from the classic quantitative genetic model conceptually in that it assumes that marker effects are random variables (Falconer and Mackay 1996; Lynch and Walsh 1998). Despite the conceptual deviation, this approach has been demonstrated to have statistically valid assumptions and applied in several studies (Verbyla *et al.* 2012; Schreck *et al.* 2019; Taylor *et al.* 2023).

ASV has several beneficial elements, making it a viable option for quantitative genetics. More importantly, it is appropriate for any quantitative discipline where variance components are of interest, from plant and microbial biology to psychology and infant research. Namely:

1. *The definitions of the variance components using ASV are additive and sum to the phenotypic variance*. Consequently, the LMM can be extended to incorporate many explanatory components, e.g. dominance, epistasis, and transcriptomic, and will yield accurate VCEs for all terms. They will sum to the total variance. This is not necessarily true for all definitions of variance components, such as the Average Marginal Variance (Piepho 2019; Feldmann, Piepho, Bridges, *et al.* 2021).
2. *ASV is well suited for stagewise analyses.* At the center of ASV is the idea that the “entry mean” is the phenotype per se, and not the observations directly. One interpretation is that individuals, not observations, are the primary source of variation or at least the primary source of interest. This concept can be easily extracted from single-stage analyses but seems at the heart of stagewise analyses (Piepho *et al.* 2012). Specifically, a single-stage analysis based on plot data can be shown to be equivalent to a stagewise analysis in which entry means and their associated variance–covariance matrix is carried forward to the second stage, in which BLUPs are computed for the genetic effects (Piepho *et al.* 2012). ASV yields accurate estimates of the genetic and genomic variance components in unreplicated or partially replicated designs common in humans, plants, and animals. ASV also yields accurate VCEs in fully efficient multistage approaches.
3. *ASV does not impact the BLUPs or breeding value predictions in Genomic (G)-BLUP.* ASV is only used to obtain accurate VCEs. It has been demonstrated that marker coding and different strategies for scaling and centering Z and K do not impact BLUPs or prediction accuracy (Strandén and Christensen 2011; Legarra 2016; Feldmann, Piepho, *et al.* 2022) and because ASV essentially works through a set of scalar coefficients determined by the experiment and population to obtain the expected features for the genomic relationship matrix. Practically, ASV does not change the information embedded in the LMM or data, only the scaling of the VCEs.
4. *ASV works under many model assumptions in GLMM analyses.* Beyond the often-assumed variance–covariance structure in this study, e.g. $R=I\sigma_{\epsilon }^{2}$, many structures will lead to nonzero covariance between entry means. ASV can be applied to designs accounting for spatial structures with auto-regressive correlations or spline-models (Rodríguez-Álvarez *et al.* 2018; Selle *et al.* 2019). ASV can also be applied to data sets where the observational units lead to nonnormality of residuals, i.e. ordinal disease scores and proportion scores (Piepho 2019).

As substantiated by our simulations in this study and the context of our previous studies, ASV with REML estimation of the underlying variance components yields accurate estimates for oligo- and polygenic effect, both individually and collectively, and BLUPs of the additive and dominance effects of marker loci (Piepho 2019; Feldmann, Piepho, Bridges, *et al.* 2021; Feldmann, Piepho, *et al.* 2022). ASV directly yields accurate estimates of genomic heritability in the observed population and can be used to adjust deviations that arise from other commonly used methods for calculating genomic relationships regardless of the population constitution, such as inbred lines and F_1_ hybrids, unstructured GWAS populations, or animal herds and flocks. We believe that $K_{ASV}$ provides a powerful approach for directly estimating genomic heritability for the observed population regardless of study organism or experiment design (Visscher *et al.* 2006, 2008, 2010). In conclusion, we recommend that genetics researchers studying humans, microbes, or (un)domesticated plants and animals consider the ASV approach.

## Data Availability

Code and output for simulations are provided in the publicly available Zenodo repository (https://doi.org/10.5281/zenodo.6981359) (Feldmann, Covarrubias-Pazaran, *et al.* 2022).
